# A Preliminary Study on Active Chitosan‐Based Edible Films Enriched With Microencapsulated Probiotics for Set‐Type Yogurt Preservation

**DOI:** 10.1002/fsn3.72129

**Published:** 2026-07-25

**Authors:** Selin Kalkan, Emine Kirkoçoğlu, Mustafa Remzi Otağ, Mehmet Soner Engin

**Affiliations:** ^1^ Department of Food Engineering Giresun University Giresun Turkey

**Keywords:** chitosan edible films, microencapsulation, probiotic delivery, shelf life

## Abstract

This study investigated the production, characterization, and application of active chitosan‐based edible films enriched with microencapsulated *Bifidobacterium animalis* ssp. *lactis* B94, *Lacticaseibacillus rhamnosus* GG, and their combination for the preservation of set‐type yogurt. The incorporation of microencapsulated probiotics significantly affected the physicochemical, mechanical, barrier, and antimicrobial properties of the films. Film thickness increased from 0.105 to 0.157 mm, moisture content ranged from 16.53% to 18.75%, and swelling index values varied between 20.63% and 32.78%. The addition of probiotics increased water vapor permeability and reduced film transparency and tensile strength. Following the drying process, probiotic survival rates ranged between 86.26% and 90.94%. The probiotic‐enriched films exhibited antimicrobial activity against *Escherichia coli* and *Staphylococcus aureus* . The developed films were applied to set‐type yogurt, and their effects on physicochemical and microbiological quality parameters were evaluated during 21 days of refrigerated storage. Yogurt samples coated with active films showed pH values between 4.02 and 5.34, titratable acidity values between 0.553% and 0.828% lactic acid, and reduced serum separation compared with control samples. Microbiological analyses of yogurt samples during storage revealed total mesophilic aerobic bacteria counts of 6.06–7.13 log CFU/g, lactic acid bacteria counts of 5.07–7.33 log CFU/g, Lactococcus spp. counts of 4.97–6.30 log CFU/g, and yeast–mold counts of 0–3.28 log CFU/g. These preliminary findings suggest that active chitosan‐based edible films enriched with microencapsulated probiotics may have potential as biologically active surface films for set‐type yogurt; however, confirmation using independent production batches is required.

## Graphical abstract

Chitosan‐based edible films incorporating microencapsulated *Bifidobacterium animalis* subsp. *lactis* B94 and *Lacticaseibacillus rhamnosus* GG were successfully developed and applied as surface coatings for set‐type yogurt. The films maintained high probiotic viability after drying and showed suitable physicochemical and antimicrobial properties. Their application preserved the microbiological quality and supported the physicochemical stability of yogurt during refrigerated storage, demonstrating their potential as active edible packaging materials.

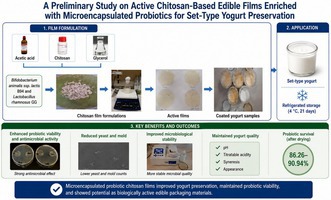

## 1 Introduction

Probiotics were first discovered by Lilly and Stillwell in 1965 (Chaudhury et al. 2015). Probiotics are defined *as live microorganisms that confer health benefits to the host when administered in adequate amounts*. These microorganisms contribute to the balance of intestinal microbiota and have been associated with various health‐promoting effects, including improved digestion, enhanced immune response, and inhibition of pathogenic bacteria (Ebrahimi et al. 2018). Today, consumer demand for healthy foods is continuously increasing, which is encouraging the development of new products in the food industry. Recent advancements have heightened researchers' interest in examining the specific characteristics of probiotic foods and their effects on human health (Pavli et al. 2018).

Researchers are working to improve the viability of probiotic microorganisms throughout food processing, storage, and consumption (De Prisco and Mauriello 2016; Burgain et al. 2011). As a result, edible polymer matrices used by the food packaging industry are being considered as an alternative for the delivery matrix of probiotic microorganisms. Nowadays, research is increasingly focusing on incorporating probiotics into biopolymeric matrices as an alternative method to enhance the health benefits of food, improve functional properties, and control pathogenic microorganisms, thus developing active/bioactive packaging materials (Coma 2008).

Recent studies have highlighted the growing interest in probiotic‐containing edible films and coatings as multifunctional food packaging systems. In addition to serving as carriers of beneficial microorganisms, these systems may contribute to food preservation by improving microbial stability and enhancing the functional value of food products (Guimarães et al. 2018; Zoghi et al. 2020). Furthermore, probiotic‐containing edible films have been proposed as promising active packaging materials due to their ability to deliver viable probiotic microorganisms while simultaneously contributing to food preservation (Espitia et al. 2016; Wai et al. 2022).

Current active packaging research aims to improve food quality and shelf life by incorporating probiotic microorganisms into edible films and coatings. Transporting probiotics through edible films and coatings not only allows for the control of viable microorganism counts but also provides protection against pH, temperature, enzymatic, and chemical changes during food production, storage, consumption, and digestion. The survival of probiotics through the gastrointestinal tract, despite low pH and bile, can also be achieved by incorporating probiotic microorganisms into edible films and coatings through microencapsulation (De Prisco and Mauriello 2016; Espitia et al. 2016).

Microencapsulation is considered one of the most effective approaches for protecting probiotic cells against environmental stresses such as oxygen exposure, acidity, moisture fluctuations, and bile salts. Encapsulation can improve probiotic survival during food processing, storage, and gastrointestinal transit, thereby enhancing their functional performance in food systems (Cook et al. 2012; De Prisco and Mauriello 2016). Therefore, the incorporation of microencapsulated probiotics into edible films may improve probiotic stability and functionality during storage and consumption.

Despite extensive research on the use of edible films and coatings in foods, only limited information is available regarding their application in yogurt systems. Kalantarmahdavi et al. (2020) reported that probiotic‐containing edible films could contribute to maintaining the microbiological quality of set yogurt during storage, while Kalantarmahdavi et al. (2022) demonstrated that protective edible layers improved yogurt shelf life and quality characteristics. However, studies evaluating the direct application of microencapsulated probiotic‐containing active chitosan films on yogurt remain scarce. Therefore, further research is needed to investigate the potential of such systems as multifunctional preservation strategies for fermented dairy products. Although yogurt is widely recognized as a functional food due to the presence of beneficial starter cultures, the viability of probiotic microorganisms may decline during storage and gastrointestinal transit. Therefore, innovative strategies are required to enhance probiotic stability during food storage. Incorporating probiotics into edible film matrices represents a promising approach to protect viable cells and improve the functional properties of food products. However, the viability of individual probiotic strains released into the final food matrix should be confirmed through strain‐specific analyses. Yogurt, a functional food, is one of the most consumed fermented dairy products in the world (Macit and Bakirci 2017). Due to its high nutritional value and health benefits, set‐type yogurt is receiving increasing interest from consumers (Qu et al. 2021). In set‐type whole‐milk yogurts, they are not homogenized due to the presence of a thin cream layer on top of the yogurt. This layer, having low permeability to oxygen, prevents the growth of undesirable microorganisms and promotes the microaerophilic growth of starter cultures, leading to higher acid production. The protective layer on yogurt is effective in enhancing the product's durability due to its antimicrobial and nutritional properties, as well as its ability to limit oxygen and prevent the ingress of spoilage agents (Kalantarmahdavi et al. 2020). This study evaluates the potential of probiotic chitosan films for improving yogurt quality. Inspired by the protective characteristics of the natural cream layer present on set‐type yogurt, the present study explores the potential application of probiotic‐containing chitosan films as surface protective systems. However, oxygen permeability of the developed films was not evaluated in the present study; therefore, direct comparisons with the oxygen barrier properties of the natural cream layer cannot be made. Although our previous study (Kalkan et al. 2025) investigated probiotic‐enriched sodium alginate films and primarily focused on their physicochemical, mechanical, barrier, and antimicrobial properties, the present study differs substantially in both formulation strategy and practical application. In the current work, chitosan was employed as the primary film‐forming polymer, while sodium alginate was used solely as an encapsulation material for probiotic incorporation. Furthermore, probiotic microorganisms were incorporated into the films in microencapsulated form rather than as free cells. Most importantly, the developed films were applied directly to set‐type yogurt, and their effects on physicochemical quality, microbial stability, and shelf‐life characteristics were comprehensively evaluated during refrigerated storage. Therefore, the present study addresses an existing research gap by evaluating the direct application of microencapsulated probiotic‐containing chitosan films on set‐type yogurt and assessing their effects on film characteristics and yogurt quality during refrigerated storage. Among the various biopolymers used in edible film production, chitosan has attracted considerable attention due to its excellent film‐forming ability, biodegradability, biocompatibility, and inherent antimicrobial activity (Martins et al. 2014; Wai et al. 2022). In addition, *Lacticaseibacillus rhamnosus* GG *and Bifidobacterium animalis* ssp. *lactis* B94 were selected because of their well‐documented probiotic properties, technological stability, resistance to gastrointestinal conditions, and widespread use in functional food applications (Kailasapathy 2006; Fritzen‐Freire et al. 2013). This study aims to determine the effects of probiotic *Lactobacillus rhamnosus* GG and *Bifidobacterium lactis* strains, microencapsulated and added to chitosan films, on the physical, chemical, and microbiological shelf life of set‐type yogurts through characterization and assessment of the bioactive properties of the prepared films.

## 2 Materials and Methods

### 2.1 Materials

In the study, the probiotic bacterial strains *Bifidobacterium animalis* ssp. *lactis* B94 and *Lacticaseibacillus rhamnosus* GG, as well as the test microorganisms *Staphylococcus aureus* ATCC 25923 and *Escherichia coli* Type 1, were obtained from the culture collection of the Department of Food Engineering, Giresun University, Giresun, Türkiye. For yogurt production, packaged fresh pasteurized milk purchased from a chain supermarket and a commercial yogurt starter culture containing *Streptococcus thermophilus* and *Lactobacillus delbrueckii* subsp. *bulgaricus* (Doğadan Bizim Maya, Istanbul, Turkey) was used for yogurt production. All chemicals used in the study are of analytical grade and were purchased from Merck Chemical Co. (Darmstadt, Germany) and Sigma‐Aldrich Co. (St. Louis, MO, USA). Chitosan (Sigma‐Aldrich, USA), with a degree of deacetylation of ≥ 75%, was used as the primary film‐forming polymer in the preparation of the edible films.

### 2.2 Preparation of Microencapsulations Containing Probiotic Microorganisms

Prior to mircoencapsulation, *Bifidobacterium animalis* ssp. *lactis* B94 and *Lacticaseibacillus rhamnosus GG* were activated twice in sterile MRS broth (Merck) at 37°C for 12 h. Activated cultures were harvested by centrifugation (5000× *g*, 10 min, 4°C), washed twice with sterile saline solution, and resuspended prior to encapsulation. Microencapsulation of probiotic microorganisms was performed using an extrusion‐based ionic gelation technique. Briefly, a sterile 2% (w/v) sodium alginate solution was prepared and mixed with the probiotic bacterial suspension under sterile conditions. The bacterial cultures were harvested by centrifugation (5000× *g*, 10 min at 4°C), washed twice with sterile saline solution, and resuspended to achieve a final concentration of approximately 10^9^ CFU/mL prior to encapsulation. The mixture was extruded dropwise through a syringe equipped with a 0.8 mm needle into a gently stirred 0.05 M calcium chloride solution, allowing gel beads to form via ionic crosslinking. The capsules were left to harden for 1 h, collected by filtration, and used for further analyses (Krasaekoopt et al. 2003).

### 2.3 Viable Cell Counts of Probiotic Microcapsules

Ten grams of microencapsulated samples were aseptically weighed using a precision balance. The samples were then transferred into 90 mL of Maximum Recovery Diluent (MRD; Merck, Germany) and homogenized for 1 min using a stomacher. Decimal dilutions were prepared, and to determine the count of *Lactobacillus rhamnosus* GG, samples were plated on MRS Agar (Merck, Germany) and incubated anaerobically at 37°C for 72 h. To determine the count of *Bifidobacterium animalis ssp lactis* B94 counts were determined on MRS agar (Merck, Germany) supplemented with 0.05% (w/v) L‐cysteine hydrochloride and incubated anaerobically at 37°C for 72 h. The results were calculated as log CFU/g (U.S. Food and Drug Administration 1998).

### 2.4 Stability of Probiotic Microcapsules Under Simulated Gastrointestinal Conditions

Free (non‐encapsulated) probiotic cells were included as control samples and subjected to the same experimental conditions to enable a comparative evaluation of the protective efficiency of alginate microencapsulation under simulated gastric and bile conditions. For simulated gastric conditions (SGC), 0.5 g of sodium chloride (NaCl) (Sigma, Aldrich) and 0.3 g of pepsin (≥ 2500 U/mg protein, Sigma, Aldrich) were added to 100 mL of distilled water and mixed using a magnetic stirrer. The pH of the mixture was adjusted to pH 2 using 1 N hydrochloric acid (Sigma, Aldrich). To assess the viability of probiotic microorganisms under SGC, 10 g of microencapsulated probiotic microorganisms were added to the prepared SGC solution and stirred for 2 h (Ünal 2014).

For bile conditions, 2 g of bile salt (Sigma, Aldrich) was added to 100 mL of MRS Broth (Merck, Germany) and mixed for 15 min using a magnetic stirrer. To assess the viability of probiotic microorganisms under bile conditions, 10 g of microencapsulated probiotic microorganisms were added to the obtained solution and stirred for 2 h (Ünal 2014).

For both conditions, *Lactobacillus rhamnosus* GG was enumerated by plating on MRS Agar (Merck, Germany) and incubating anaerobically at 37°C for 72 h. *Bifidobacterium animalis ssp lactis* B94 counts were determined on MRS agar (Merck, Germany) supplemented with 0.05% (w/v) L‐cysteine hydrochloride and incubated anaerobically at 37°C for 72 h Results were calculated as log CFU/g (U.S. Food and Drug Administration 1998).

### 2.5 Preparation of Active Chitosan Films

The method used for preparing chitosan films was modified from that of Sánchez‐González et al. (2013). For film production, 1 mL of acetic acid (Sigma, Aldrich) was added to 100 mL of distilled water and dissolved using a magnetic stirrer. To the prepared 1% acetic acid solution, 1 g of chitosan (Sigma, Aldrich) was added and stirred at 80°C for 1 h. Subsequently, 0.75 mL of glycerol (20%, v/v; Merck, Germany) was added as a plasticizer, and heating was stopped, continuing to stir the solution for an additional 10 min. Once the temperature reached 37°C, 1 g of microencapsulated probiotic microorganisms (approximately 10^8^ CFU/g) was added to the film solution to ensure that the final viable cell count in the dried films remained above the recommended minimum level for probiotic functionality, and the active film solutions were stirred using a magnetic stirrer for 30 min. Sodium alginate was used exclusively for the microencapsulation of probiotic microorganisms through ionic gelation with calcium chloride (CaCl_2_) and was not employed as a film‐forming polymer in the preparation of chitosan films. Therefore, the final film matrix consisted primarily of chitosan, while alginate was present only as a component of the incorporated probiotic microcapsules. Considering the formulation used, the amount of alginate present in the final dried films was considered to be limited and originated solely from the incorporated alginate–CaCl_2_ microcapsules. After stirring, the active film solutions were poured into sterile glass Petri dishes (9.5 cm diameter) in 12.5 mL portions, and the Petri dishes were left to dry at 37°C for 12 h, resulting in the formation of films containing microencapsulated probiotic microorganisms. The films were stored in a desiccator at 25°C and 50% ± 4% RH for 72 h for subsequent analysis. The produced films were labeled as follows: C: for chitosan films without probiotic microorganisms, C‐B: for chitosan films containing *Bifidobacterium lactis* , C‐L: for chitosan films containing *Lactobacillus rhamnosus* GG, and C‐BL: for chitosan films containing both *Bifidobacterium lactis* and *Lactobacillus rhamnosus* GG. Yogurt samples coated with films were coded as follows: C‐Y (control yogurt), C‐B‐Y, C‐L‐Y, and C‐BL‐Y. The enumeration of probiotic microorganisms in the films was determined according to the method of Ceylan and Atasoy (2022). The viability of probiotic microorganisms after film drying was calculated by comparing the viable cell counts in the film‐forming solutions before drying and in the corresponding dried film samples after drying. Microbial counts were expressed as log CFU/g of sample. Viability (%) was calculated according to the method of Ceylan and Atasoy (2022) based on the survival of viable cells after drying relative to the initial viable cell counts before drying. All film‐forming solutions were prepared under aseptic conditions. Prior to probiotic incorporation, the solutions were sterilized, cooled to room temperature, and the microencapsulated probiotics were added aseptically to minimize contamination during film preparation.

### 2.6 Antimicrobial Properties of Active Films

The antimicrobial activity of the prepared chitosan film samples against the pathogenic bacteria *Staphylococcus aureus* ATCC 25923 and *Escherichia coli* Type 1 was determined using the Disk Diffusion method. Disks with a diameter of 6 mm were cut from the active chitosan films prepared with different compositions. Freshly cultured microorganisms were incubated in liquid medium until a concentration of 10^6^ CFU/mL was reached and then plated on Mueller‐Hinton Agar. The disks cut from the active chitosan films were placed on the inoculated Petri dishes, which were then incubated at 37°C for 24 h. After incubation, the inhibition zone diameters around the disks were measured in millimeters. Chitosan films without probiotic incorporation were used as negative controls to evaluate the contribution of probiotic microorganisms to the antimicrobial activity of the films. No antibiotic‐based positive control was included, as the primary objective was to compare probiotic‐enriched films with the corresponding control film formulation. Each analysis was conducted in triplicate.

### 2.7 Physicochemical Properties of Active Films

Thickness measurements of the films were conducted using a precision micrometer (Mitutoyo Digimatic Micrometer, Model 293‐831‐30, Mitutoyo Corp., Kawasaki, Japan) with an accuracy of 0.001 mm, with each film measured at six different points, and the average thickness was calculated (Engin et al. 2022). The moisture content of the films was determined using a modified method from Bakry et al. (2017). According to this method, 2 × 2 cm film samples were weighed using a precision balance and dried at 105°C until a constant weight was achieved. The moisture content was then determined using the following equation (Equation 1).

(1) $$C\;\left(\%\right)=\left[\frac{m1-m2}{m1}\right]\times 100$$

In the equation, C represents the % moisture content of the film samples, *m*1 denotes the initial weight of the film samples, and *m*2 denotes the final weight of the film samples after drying.

Film densities were calculated based on the direct weights and dimensions of the films, using the following equation (Equation 2) (Ramos et al. 2012).

(2) $$\rho s=\text{𝑚/}\left(\text{𝐴}\times \gamma \right)$$

*ρ*s: Dry matter density (g/cm^3^), m: Dry mass weight (g), A: Film surface area (cm^2^), γ: Film thickness (cm).

Film hydrophilicity/hydrophobicity was evaluated by contact angle measurements. For this purpose, a goniometer (Dataphysics OCA 15EC) was used, where a droplet was placed on the film surface and tested using the sessile drop technique. Measurements were taken at 25°C on each film. The swelling index was determined by immersing 2 × 2 cm film samples in distilled water at 25°C. Afterward, excess water on the films was removed with a tissue, and the films were weighed again. The amount of absorbed water was calculated as a percentage. For color measurements of the film samples, L* (lightness), a* (red‐green), and b* (yellow‐blue) color parameters were measured using a HunterLab ColorFlex EZ colorimeter (Hunter Associates Laboratory Inc., Reston, VA, USA) on a standard white background. Color changes (ΔE*) of the films were calculated using the following equation (Equation 3). Each measurement was repeated three times (Engin et al. 2022).

(3) $$\mathrm{\Delta E}*={\left[{\left(\mathrm{Lo}*-L*\right)}^{2}+{\left(\mathrm{ao}*-a*\right)}^{2}+{\left(\mathrm{bo}*-b*\right)}^{2}\right]}^{1/2}$$

Optical and UV barrier properties were evaluated using UV–Vis spectrophotometry. The absorbance spectra (200–800 nm) for each sample were recorded using a Hach Lange GmbH spectrophotometer (Germany). The standard test method ASTM D1003‐00 was used for light transmittance. The opacity of the films (AU nm) was determined according to the method of López and García (2012). Film transparency was calculated as the ratio between absorbance at 600 nm (A600) and film thickness, and expressed as A600/mm.

### 2.8 Mechanical and Barrier Properties of Active Films

The tensile strength (TS) and percentage elongation of the active film samples were determined using the average measurements of 10 film samples cut to dimensions of 80 × 25 mm, according to ASTM‐D638 (ASTM 2000), with a TA‐TX2 (Texture Expert Exceed 2.3, Stable Micro System, Surrey, UK). Each film strip was placed in pneumatic jaws (25 psi) and stretched at a starting distance of 1 mm/s between 100 mm jaws (Kalkan et al. 2020).

The water vapor permeability (WVP) values of the active film samples were determined gravimetrically at 25°C ± 1°C using a modified ASTM E96‐80 (ASTM 1995) procedure. Film samples were placed in Delrin containers containing 2 g of anhydrous CaCl_2_ (Merck, Darmstadt, Germany) at 0% RH, and the containers were then placed in desiccators containing BaCl_2_ (75% RH) in incubators at 25°C. The weight of the test containers was measured every 2 h over a 12‐h period. The water vapor transmission rates (WVTR) and water vapor permeability values (WVP) of the film samples were calculated using Equations (4) and (5).

(4) 𝑊𝑉𝑇𝑅=𝛥𝑚/(𝐴×𝑡)

(5) 𝑊𝑉𝑃=𝑊𝑉𝑇𝑅×𝑑/𝛥𝑃

where Δ*m* is the weight change before and after the test (g); *t* is the test duration (h); A is the test area (m^2^); *d* is the film thickness (mm); and Δ*P* is the partial pressure difference between the films (kPa) and was calculated from the difference in water vapor partial pressures between the two sides of the film under the experimental relative humidity conditions (Engin et al. 2022).

### 2.9 Scanning Electron Microscopy of Active Films

The surface morphology of the film samples was examined using a scanning electron microscope (Nova NanoSEM 200, FEI, USA). Prior to imaging, the samples were mounted on aluminum stubs and coated with a thin layer of gold. SEM observations were performed at an accelerating voltage of 10 kV. At least six surface micrographs were obtained from each film sample at different magnifications. No cross‐sectional SEM images were acquired in the present study.

### 2.10 Fourier Transform Infrared (FTIR) Spectroscopy Analysis of Active Films

The FTIR spectra of the films were obtained using a Jasco 6600 spectrometer (Jasco Corporation, Tokyo, Japan). Each spectrum was recorded with 10 scans in the range of 4000 cm^−1^ and a resolution of 4 cm^−1^. The results were then used to determine the interactions between functional groups of the polymer‐glycerol and the active compound (Maizura et al. 2007).

### 2.11 Thermogravimetric Analysis (TGA) of Active Films

The thermal properties of the films were determined using a Seteram Instrumentation Labsys Evo (Caluire, France). For the analysis, 10 mg of film samples were weighed and placed in airtight aluminum containers, then heated in an argon atmosphere from 0°C to 790°C at a rate of 10°C/min.

### 2.12 Yogurt Production and Film Application

For the production of yogurt samples, pasteurized daily milk was heated to the fermentation temperature. Using a yogurt starter culture, fermentation was carried out under the specified conditions. The fermented samples were then transferred to 400 mL sterile glass jars and incubated at 42°C for 8 h. After incubation, to solidify the structure, the yogurt samples were cooled to 4°C under refrigeration conditions. Approximately 350 g of yogurt was placed into each sterile glass jar. Active chitosan film samples were cut according to the diameter of the glass jars and aseptically applied to completely cover the yogurt surface. Yogurt samples without film application were used as the control group. All samples were stored at 4°C and analyzed on days 0, 7, 14, and 21 of storage.

### 2.13 Analysis of Film‐Coated Yogurt Samples

The total solids and ash were determined by gravimetric method. Syneresis was determined by the methods described by Macit and Bakirci (2017). The pH values were measured using a digital pH meter (Five Easy Plus pH meter FP20, MettlerToledo, USA). For Brix and density analysis, an Anton Paar (DMA 500) analyzer was used. First, 40 g of yogurt samples were weighed and filtered using filter paper to obtain a homogeneous sample. The separated serum was injected into the sample intake section of the device, and the displayed data were recorded. To determine titratable acidity, expressed as a percentage of lactic acid, 10 g of yogurt was mixed with 20 mL of distilled water and titrated with 0.1 N NaOH using phenolphthalein as an indicator until a faint pink color was achieved (Kalkan et al. 2018). Water activity values were measured using a water activity device (Aqualab 4TE, METER Group Inc. USA). The samples at room temperature were placed in the sample compartment of the device for measurement. The values displayed on the screen were recorded. The color of yogurt was measured using Hunterlab Colorimetre. The colorimeter used L* (lightness), a* (redness), and b* (yellowness) scales (Macit and Bakirci 2017).

Bacterial counts were performed on the 0th, 7th, 14th, and 21st days in triplicate for each batch. For microbiological analyses, yogurt samples were homogenized together with the edible film layer in order to evaluate the overall microbiological contribution of the probiotic‐containing film system during storage. Samples (10 mL) were diluted with ringer solution (90 mL). Subsequently, serial dilutions were made, and colonies were counted. Plate Count Agar (PCA; Merck, Germany, incubated at 37°C for 24 h) was used for the total mesophilic aerobic bacteria count, Man, Rogosa and Sharpe Agar (MRS Agar; Merck, Germany, incubated at 37°C for 72 h under anaerobic conditions) for the total lactic acid bacteria (LAB) count, M17 Agar (Merck, Germany, incubated at 37°C for 24 h) for *Lactococcus* spp. count. Total yeast and mold counts were determined on acidified Potato Dextrose Agar (PDA; Merck, Germany) incubated at 30°C for 5 days.

### 2.14 Statistical Analysis

For film characterization analyses, including physicochemical, mechanical, barrier, structural, and thermal properties, data were analyzed using IBM SPSS Statistics 19.0 software. One‐way analysis of variance (ANOVA) followed by Duncan's multiple range test was applied where appropriate, and differences were considered significant at *p* < 0.05. Data were expressed as mean ± standard deviation (SD) of three technical replicates. For antimicrobial activity and yogurt application experiments, the results were obtained from technical replicates of a single production batch rather than independent biological or process‐level replicates. Therefore, these data were reported descriptively as mean ± standard deviation (SD), and no inferential statistical comparisons were performed for Tables 6 and 7, Figure 5.

## 3 Results and Discussion

### 3.1 Viability of Probiotic Microcapsules

Table 1 presents the viability levels of microencapsulated *L. rhamnosus* and *B. lactis* strains following the microencapsulation process intended for the preparation of active chitosan film samples. *B. lactis* exhibited higher viability after encapsulation than *L. rhamnosus* . Previous studies have demonstrated that the viability of probiotic microorganisms during microencapsulation is strongly influenced by the type and concentration of encapsulating materials, processing conditions, and drying treatments applied during production (Ünal 2014; Popović et al. 2021; Wai et al. 2022). Based on the obtained results, the viable cell counts detected after the encapsulation process remained within the expected range for probiotic microencapsulation systems. These findings suggest that the encapsulation procedure effectively protected probiotic cells and minimized viability losses during processing. Similar survival rates have been reported for alginate‐based encapsulation systems and probiotic‐containing edible films, where probiotic viability remained at levels suitable for functional food applications (Akman et al. 2021; Abedinia et al. 2021; Zoghi et al. 2020).

**TABLE 1:** Probiotic viable cell count in microcapsules (log CFU/g).

Microorganisms	Microencapsulated viable cell count
*B. lactis*	9.50 ± 0.45^a^
*L. rhamnosus*	8.05 ± 0.21^b^

*Note:* ^a,b^Different superscripts in the same column indicate a significant difference at *p* ≤ 0.05 level. It indicates that the values are expressed as mean ± standard deviation (SD) and provides the definitions of the sample abbreviations (C, C‐B, C‐L, and C‐BL).

### 3.2 Survival Under Simulated Gastrointestinal Conditions

Table 2 presents the viability of microencapsulated probiotic microorganisms under simulated gastric and bile conditions. Exposure to these stress conditions reduced the initial probiotic counts from approximately 8–9 log CFU/g to 6–7 log CFU/g. Furthermore, *B. lactis* exhibited slightly higher survival than *L. rhamnosus* under both simulated gastric and bile conditions, suggesting a greater resistance to gastrointestinal stress. To demonstrate the beneficial health effects of LABs, they must withstand the stressful conditions of bile‐containing upper intestines. The results showed that the viable cell counts of encapsulated probiotic microorganisms decreased after exposure to simulated gastric and bile conditions but remained within the detectable range. These findings suggest that alginate‐based encapsulation may contribute to maintaining probiotic viability under simulated gastrointestinal conditions. These findings are consistent with those reported by Chandramouli et al. (Chandramouli et al. 2004) and Kailasapathy (Kailasapathy 2006). Similarly, Fritzen‐Freire et al. (Fritzen‐Freire et al. 2013) examined the effects of artificial stomach conditions on free and microencapsulated bifidobacteria at 5 and 10 g/L bile salt concentrations and found that microencapsulations provided the highest survival rates of bifidobacteria compared to free cells after exposure to artificial stomach conditions.

**TABLE 2:** Resistance of microencapsulated probiotic microorganisms to bile salts and simulated gastric conditions (log CFU/g).

Mikroorganizma	Bile salts	Simulated gastric conditions
*B. lactis*	6.80 ± 0.16^a^	7.01 ± 0.08^a^
*L. rhamnosus*	6.21 ± 0.20^b^	6.09 ± 0.15^b^

*Note:* ^a,b^Different superscripts in the same column indicate a significant difference at *p* ≤ 0.05 level. It indicates that the values are expressed as mean ± standard deviation (SD) and provides the definitions of the sample abbreviations (C, C‐B, C‐L, and C‐BL).

### 3.3 Viability of Probiotics in Active Films

The most important steps for the survival of probiotics in edible films are reported to be the film casting and drying processes. In film production, high drying stability of probiotic microorganisms is considered an important criterion for evaluating probiotic films (Kalantarmahdavi et al. 2020). The preparation of probiotic‐containing films involves two harmful steps for probiotic bacteria: the osmotic effect of the film‐forming solution and the dehydration effect of drying (Romano et al. 2014). Given the drying conditions of the film‐forming solution, osmotic stress‐induced damage to the cytoplasmic membrane is thought to be responsible for the reduction in probiotic microorganism numbers (Fu and Chen 2011). Therefore, many researchers have investigated the viability of bacterial cells during both the drying process and storage period in polysaccharide‐based films such as pectin, cellulose, or alginates (Yonekura et al. 2014; Cook et al. 2012; Ceylan and Atasoy 2022; Bustos and Bórquez 2013; Soukoulis et al. 2014).

**FIGURE 1:**
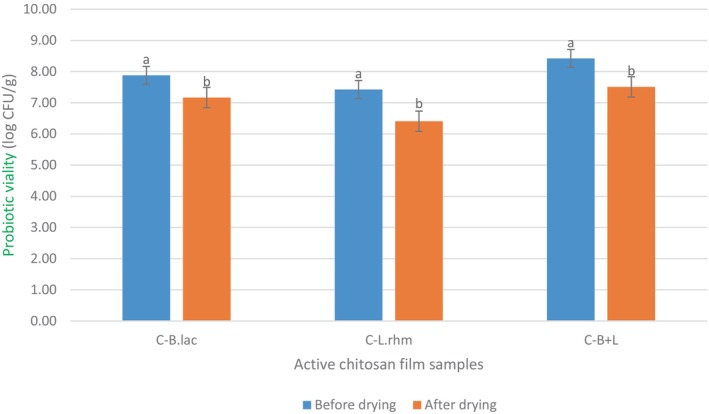
Viability of probiotic microorganisms in active chitosan films (log CFU/g; Different lowercase letters indicate significant differences between samples (*p* < 0.05); C‐L.rhm: chitosan film containing *L. rhamnosus* GG; C‐B.lac: chitosan film containing *B. lactis* B94; C‐B+L: chitosan film containing both probiotic strains).

As shown in Figure 1, the number of viable probiotic cells in the films decreased after drying (*p* ≤ 0.05). The viability values of probiotic microorganisms in the films after drying range from 86.26% to 90.94%. These results indicate that the encapsulation and film‐forming processes provided adequate protection against drying‐induced stress and enabled the maintenance of high probiotic viability. Similar viability values have been reported in studies evaluating probiotic survival in polysaccharide‐based edible films and encapsulation systems (Cook et al. 2012; Ceylan and Atasoy 2022). Similarly, in a study by Ceylan and Atasoy (2022), the viability values of *B. animalis subsp. lactis* BB‐12 were found to be between 86.73% and 93.38% after drying. The viability values obtained in the present study fall within this range, indicating that the developed chitosan‐based film system effectively preserved probiotic viability during drying. Despite the absence of prebiotic compounds in the formulations, the developed films maintained high probiotic survival rates, suggesting that microencapsulation combined with the chitosan matrix provided sufficient protection against dehydration stress.

### 3.4 Physicochemical Properties of the Films

The thickness, hydration properties (moisture, density, contact angle), and color properties of active film samples enriched with microencapsulated probiotic microorganisms are presented in Table 3.

**TABLE 3:** Some physicochemical properties of active film samples.

Film samples					
	Parameters	Control film (C)	C‐B	C‐L	C‐BL
Thickness (mm)		0.112 ± 0.00^c^	0.105 ± 0.01^c^	0.133 ± 0.02^b^	0.157 ± 0.01^a^
Hydration properties	Moisture (%)	16.53 ± 1.03^c^	17.73 ± 0.10^b^	17.13 ± 0.02^b^	18.75 ± 1.48^a^
	Density (g cm^−3^)	1.14 ± 0.24^a^	1.29 ± 0.07^a^	0.90 ± 0.24^ab^	0.71 ± 0.16^c^
	Contact angle (θ°)	84.47 ± 0.45^a^	75.77 ± 4.06^b^	70.60 ± 17.88^b^	57.02 ± 2.22^c^
	Swelling index (%)	20.63 ± 0.01^c^	24.59 ± 0.17^b^	26.84 ± 0.01^b^	32.78 ± 1.01^a^
Color properties	L*	87.06 ± 0.34^ab^	87.19 ± 0.32^ab^	87.64 ± 0.89^a^	86.86 ± 0.22^a^
	a*	−1.02 ± 0.15^c^	−0.88 ± 0.03^ab^	−0.65 ± 0.34^b^	−0.43 ± 0.19^a^
	b*	6.14 ± 1.17^a^	5.00 ± 0.12^b^	3.62 ± 1.71^c^	4.21 ± 0.99^bc^
	ΔE*	12.22 ± 0.53^a^	12.16 ± 0.25^ab^	11.66 ± 0.48^c^	12.31 ± 0.19^a^

*Note:* ^a–c^Different superscripts in the same row indicate a significant difference at *p* ≤ 0.05 level; C: chitosan film without probiotic microorganisms; C‐B: chitosan film containing Bifidobacterium lactis; C‐L: chitosan film containing Lacticaseibacillus rhamnosus GG; C‐BL: chitosan film containing both *Bifidobacterium lactis* and *Lacticaseibacillus rhamnosus* GG. It indicates that the values are expressed as mean ± standard deviation (SD) and provides the definitions of the sample abbreviations (C, C‐B, C‐L, and C‐BL).

The thickness of the films is an important criterion to consider when selecting appropriate packaging types and directly affects the solubility and mechanical properties of the packaging films (Wai et al. 2022). As shown in Table 3, the thickness values of the active film samples range from 0.105 to 0.157 mm. The lowest thickness value was found in the film samples enriched with *B. lactis* , while the highest thickness values were observed in the film samples enriched with *B. lactis* and *L. rhamnosus* . A significant difference in thickness was observed between the control group and the probiotic‐containing edible films (*p* ≤ 0.05). The increase in film thickness observed in probiotic‐containing samples may be attributed to the incorporation of microcapsules into the film matrix, which increased the total solid content of the film‐forming solution. Similar observations have been reported in probiotic edible film systems where the addition of encapsulated cells resulted in thicker film structures (Roshandel‐Hesari et al. 2022). With the increase in the amount of sodium alginate used in the microencapsulation of the film structure and the density of the added probiotic microorganisms, the film thickness also increased. Higher total solids content in the film‐forming solution leads to greater film thickness. Similarly, Soukoulis et al. (2014) reported that the addition of probiotic bacterial cells to film‐forming solutions altered the film thickness. The increase in film thickness may also be associated with the physical incorporation of alginate microcapsules into the chitosan matrix, resulting in a less compact structure and greater solid content within the film network (Roshandel‐Hesari et al. 2022; Wai et al. 2022).

The moisture content of the film samples after drying not only affects the rate of viability reduction of probiotic microorganisms during long‐term storage but also facilitates the dissolution of edible films in the mouth (Kanmani and Lim 2013). Therefore, it is important to quantitatively determine the moisture content in the films. As shown in Table 3, the moisture values of the active film samples ranged from 16.53% to 18.75%. A statistically significant increase was observed between the control films and the microencapsulated film samples (*p* ≤ 0.05). The mass transfer of water vapor from the film matrix is the driving force of moisture dispersion. The balanced moisture levels in edible films are influenced by polymer concentration, the amount and type of plasticizer, and water retention capacity (Abedinia et al. 2021). The increase in moisture content in active chitosan films may be attributed to the individual water retention capacity of sodium alginate used in the microencapsulation process and its interactions with the plasticizer (Phovisay et al. 2018). The hygroscopic nature of sodium alginate can help retain water within the film matrix (Verde et al. 2021). Furthermore, the incorporation of alginate‐based microcapsules may have increased the availability of hydrophilic sites within the film network, thereby enhancing water retention capacity. Additionally, the increase in film thickness with the addition of microencapsulated probiotics has likely partially restricted moisture loss from the film (Jansson and Thuvander 2004; Wai et al. 2022). As seen in Table 3, the density values of the films ranged from 0.71 to 1.41 g/cm^3^. However, with the addition of microencapsulated probiotics to the films, there was a significant decrease in density values (*p* ≤ 0.05). The thickness and density of active chitosan films are related to the type of biopolymer used in production, the additives employed, and their concentrations (Karimi et al. 2020).

The water contact angle is measured to predict potential interactions between food and packaging materials. As shown in Table 3, the contact angle values of the films ranged from 57.02° ± 2.22° to 84.47° ± 0.45°. In our study, the highest contact angle value was obtained with the control films. Similarly, Sultan et al. (2021) measured the contact angle of chitosan films as 79.8°, indicating that the surface is partially hydrophobic due to the presence of hydroxyl groups in the chitosan/glycerol mixture. The addition of microencapsulated probiotic microorganisms to the films caused a significant change in the contact angle values (*p* ≤ 0.05). Generally, a low contact angle (TA < 65°) indicates a hydrophilic surface, while a high contact angle (TA > 65°) signifies hydrophobic surfaces (Santos et al. 2017). According to the analysis results, the hydrophilic character of the control films became hydrophobic with the addition of microencapsulated probiotic microorganisms. de Oliveira et al. (2021) found in a similar study that the addition of prebiotic fructooligosaccharides to the film likely reduced the film's hydrophilicity by interacting with bacterial surface polysaccharides.

Swelling is one of the most important properties determining the use of chitosan films in packaging applications. As shown in Table 3, the swelling index (%) values of the films ranged from 20.63 ± 0.01 to 32.78 ± 1.01. The highest swelling index was observed in films enriched with *B. lactis* and *L. rhamnosus* , while the lowest values were found in the control films. Chitosan is hydrophilic, and since water diffuses into chitosan more rapidly, the films start to swell before dissolving. Therefore, during the initial stages of hydration, bond breaking and dissolution of chitosan also occur, although swelling happens before dissolution (Ren et al. 2005). The differences observed in swelling index results among the films may be attributed to the effects of microorganisms added to the film components on parameters such as the nature of the polymer, polymer chain flexibility, molecular weight, crystal structure, and chemical composition (Taghizadeh and Davari 2006). Our study results are consistent with those of Bhuvaneshwari et al. (2011), who reported a swelling index ranging from 10% to 49%.

The color characteristics of edible films are crucial for their use in packaging, as they significantly impact the overall appearance and consumer acceptance (Khoshgozaran‐Abras et al. 2012). As shown in Table 3, the L* value (lightness) of the films ranged from 86.86 ± 0.22 to 87.64 ± 0.84; the a* value (redness/greenness) ranged from −0.43 ± 0.19 to −1.02 ± 0.15; the b* value (yellowness/blueness) ranged from 3.62 ± 1.71 to 6.14 ± 1.17; and the ∆E* value (total color difference) ranged from 11.66 ± 0.48 to 12.31 ± 0.19. These results indicate that the addition of probiotics led to a reduction in the films' clarity (L* value), although this decrease was not statistically significant. Probiotic‐containing films were found to have higher a* values (greenness) and ∆E* values (total color difference). Thus, it can be said that probiotic microorganisms impart a greenish tint to the films, though this was not confirmed by visual observations. The b* value (yellowness) of the films was affected by the addition of probiotic microorganisms, showing a decrease.

Transparency in edible films is an important characteristic as it reflects the sensory qualities of food products and can increase consumer acceptance of transparent polymeric packaging materials (Wai et al. 2022). The UV light absorption of biodegradable films is significant, as this radiation can catalyze oxidative degradation, potentially negatively impacting the shelf life of especially fatty foods (López and García 2012). Table 4 shows the light transmittance and transparency values of probiotic‐enriched chitosan films and a synthetic film in the wavelength range of 200–800 nm. At 200 nm, the synthetic film has very low transmittance, whereas the probiotic‐enriched films do not transmit UV‐C radiation. The transmittance value at a wavelength of 280 nm is quite high for the synthetic film, but the transmittance values for the control films and probiotic‐enriched films are significantly different from those of the synthetic film, indicating that UV‐B radiation is largely absorbed. A similar trend continues at 350 nm, and it is observed that approximately 60% of UV‐A radiation is absorbed. Probiotic‐enriched films exhibit significant barrier properties against UV radiation, suggesting that they can prevent photoxidation caused by UV light to some extent. It is observed that probiotic‐enriched films absorb approximately 30% of the visible light radiation at UV 400 nm, while absorbance significantly decreases between 500 and 800 nm. Although probiotic‐enriched films show lower transparency values compared to synthetic films, these results indicate that the films could be used as transparent packaging or coating materials.

**TABLE 4:** Transparency values of film samples.

Light transmittance of film samples (%) and transparency values (A600/mm)									
Samples	At different wavelengths (nm) transparency values (%) Transparency values								
	200	280	350	400	500	600	700	800	(A600/mm)
LDPE	5.7	76.4	82.0	83.9	86.2	87.5	88.2	89.0	2.07
Control (C)	0	21.5	41.5	66.6	80.4	84.5	88.2	87.6	0.65
C‐B+L	0	26.5	48.9	72.0	84.4	87.0	88.1	88.6	0.40
C‐B.lac	0	23.4	45.5	70.9	83.7	87.1	87.6	87.7	0.57
C‐L.rhm	0	19.9	39.1	66.6	81.0	84.2	86.3	87.1	0.57

*Note:* C: chitosan film without probiotic microorganisms; C‐B: chitosan film containing *Bifidobacterium lactis* ; C‐L: chitosan film containing Lacticaseibacillus rhamnosus GG; C‐BL: chitosan film containing both *Bifidobacterium lactis* and *Lacticaseibacillus rhamnosus* GG. It indicates that the values are expressed as mean ± standard deviation (SD) and provides the definitions of the sample abbreviations (C, C‐B, C‐L, and C‐BL).

### 3.5 Mechanical and Barrier Properties of Films

Edible films must be sufficiently resistant to external factors to be used as food packaging materials. Additionally, these films should be flexible and durable during packaging and storage (Pranoto et al. 2005). Tensile strength (TS) and elongation percentage (E, %) are two fundamental indicators of the mechanical properties of edible films used for packaging. The mechanical properties of the film samples are summarized in Table 5. As shown in Table 5, the addition of probiotic cells to glycerol‐plasticized chitosan films significantly reduced tensile strength (*p* ≤ 0.05). The reduction in tensile strength may be attributed to discontinuities created by the incorporated microcapsules within the chitosan matrix, which can weaken intermolecular interactions between polymer chains and reduce film cohesion. Similar effects have been reported in probiotic‐containing edible films, where the incorporation of encapsulated particles altered the structural integrity of the polymer network (Wai et al. 2022). This result is consistent with the findings of Kanmani and Lim (2013), who observed that the incorporation of bacterial cells into pure pullulan films decreased TS. However, Gialamas et al. (2010) reported that the mechanical properties of sodium caseinate films were not affected by bacterial cells. In our study, the addition of probiotics to the film matrix led to a notable decrease in elongation at break, indicating reduced film flexibility. This reduction may be associated with restricted mobility of the chitosan polymer chains due to the presence of microcapsules, which can limit the ability of the film matrix to deform under stress. The control film samples had the highest E (%) values, while the lowest E (%) values were found in the C‐L.rhm film samples (*p* ≤ 0.05).

**TABLE 5:** Mechanical and barrier properties of film samples.

Film samples	Mechanical and barrier properties		
	Tensile strength (Mpa)	Elongation at break (E; %)	Water vapor permeability (WVP; 10^−12^ g cm/cm^2^ s Pa)
Control (C)	15.37 ± 3.09^a^	74.58 ± 0.88^a^	2.13 ± 0.00^b^
C‐B.lac	10.10 ± 3.80^c^	66.13 ± 0.90^ab^	2.12 ± 0.00^b^
C‐L.rhm	11.32 ± 3.18^b^	47.40 ± 0.75^c^	2.64 ± 0.01^ab^
C‐B+L	11.10 ± 2.60^b^	62.20 ± 0.66^b^	2.93 ± 0.05^a^

*Note:* ^a–c^Different superscripts in the same column indicate a significant difference at *p* ≤ 0.05 level. C: chitosan film without probiotic microorganisms; C‐B: chitosan film containing *Bifidobacterium lactis* ; C‐L: chitosan film containing Lacticaseibacillus rhamnosus GG; C‐BL: chitosan film containing both *Bifidobacterium lactis* and *Lacticaseibacillus rhamnosus* GG. It indicates that the values are expressed as mean ± standard deviation (SD) and provides the definitions of the sample abbreviations (C, C‐B, C‐L, and C‐BL).

Biopolymers are generally sensitive to moisture absorption. Water vapor permeability (WVP) is one of the most critical properties of edible films and can be influenced by factors such as film structural integrity, hydrophobic ratio, crystalline and amorphous content, and thickness (Kanmani and Lim 2013). The WVP value of films is important for preventing the mass transfer of food to the surrounding environment (Bertuzzi et al. 2007; Kristo and Biliaderis 2006). As shown in Table 5, the addition of probiotics increased the WVP of chitosan films compared to the control group. However, no significant difference was observed between different probiotic films (*p* ≤ 0.05). The increase in WVP in probiotic‐containing films may be attributed to structural heterogeneity created by the presence of microcapsules in the polymer matrix. These structural discontinuities may facilitate water vapor diffusion through the film network. Differences among the results can be attributed to variations in biopolymer types and film preparation methods.

### 3.6 Structural Properties of Films

**FIGURE 2:**
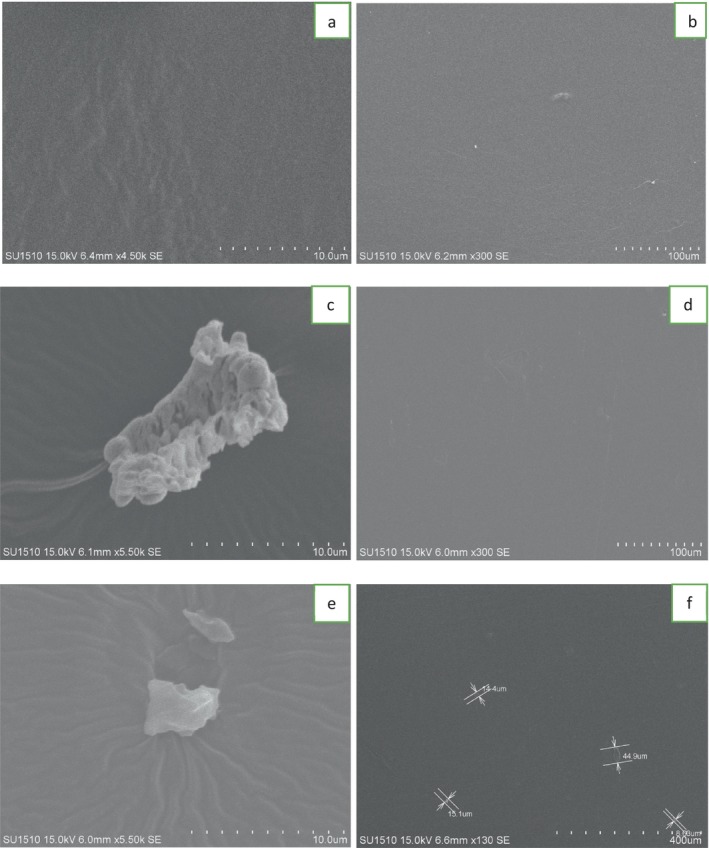
SEM micrographs of chitosan film samples (a) control chitosan film; (b) chitosan film containing microencapsulated *L. rhamnosus* GG; (c) higher‐magnification view showing *L. rhamnosus* microcapsules embedded in the film matrix; (d) chitosan film containing microencapsulated *B. lactis* B94; (e) higher‐magnification view showing *B. lactis* microcapsules embedded in the film matrix; (f) chitosan film containing both microencapsulated probiotic strains.

Scanning electron microscopy (SEM) was used to analyze the effects of adding microencapsulated probiotic microorganisms on the morphological structure of chitosan films. The obtained SEM images are shown in Figure 2. As seen in Figure 2, the chitosan film samples exhibited a smooth surface without cracks or pores. Examination of the SEM images indicates that the addition of microencapsulated probiotics to the chitosan films resulted in good integration of bacterial cells into the film matrix (small, round‐like shapes), thereby enhancing the protective effect of the bacterial cells within the films. Additionally, the presence of more microencapsulated cells could be responsible for the higher water vapor permeability (WVP) values observed in the films. The rougher surface morphology observed in probiotic‐containing films is consistent with the incorporation of microcapsules into the chitosan matrix and may explain the increased thickness and water vapor permeability values observed in these samples, as the embedded particles can disrupt the continuity of the polymer network and create additional diffusion pathways for water vapor (Akman et al. 2021; Popović et al. 2021). The increase in WVP may enhance the solubility of the films and indirectly improve the release of probiotics (Akman et al. 2021). Similar to our results, Pereira et al. (2016) and Ebrahimi et al. (2018) reported that the addition of probiotic microorganisms did not result in a highly porous structure in the films.

**FIGURE 3:**
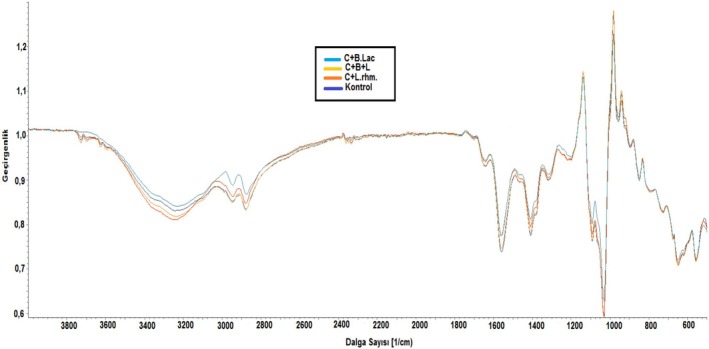
FT‐IR spectra of active chitosan films.

The FT‐IR spectra of the films produced from chitosan, sodium alginate, glycerol, and probiotic lactic acid bacteria are shown in Figure 3. The broad absorption band observed around 3600–3200 cm^−1^ indicates the stretching vibrations of free, inter‐molecular, and intra‐molecular hydroxyl groups (Oun and Rhim 2015). This situation can be attributed to the stretching vibrations of hydroxyl groups in chitosan, sodium alginate, and glycerol. The weak shoulder of the film structure shows the CH stretching vibrations of aliphatic —CH groups at 2950 and 2850 cm^−1^. Additionally, peaks for chitosan and sodium alginate are observed at 1600 and 1400 cm^−1^, respectively, while the asymmetric stretching peaks of carboxylate salt groups are seen at 1300 cm^−1^ (C—O stretching), 1100 cm^−1^ (C—C stretching and C—O stretching), and 950 cm^−1^ (C—O stretching) (Brugnerotto et al. 2001; Sartori et al. 1997). The 1600 cm^−1^ region corresponds to the amide region for microorganisms, while the 1400 cm^−1^ region, known as the mixed region, is where peaks for carboxyl groups in proteins, free amino acids, polysaccharides, fatty acids, and phosphate‐containing components are observed. The weak shoulder around 1300 cm^−1^ could be due to the C—O stretching vibrations from sodium alginate and glycerol, while the weak shoulder observed between 1250 and 1200 cm^−1^ may be attributed to RNA/DNA and phospholipid sources from microorganisms. The strong peak around 1100 cm^−1^ is likely due to C—C and C—O stretching vibrations. This region can also be associated with polysaccharides for microorganisms, where fingerprint‐like absorption bands of carbohydrates in the cell wall may be observed (Kılıç and Karahan 2010; Engin et al. 2022).

**FIGURE 4:**
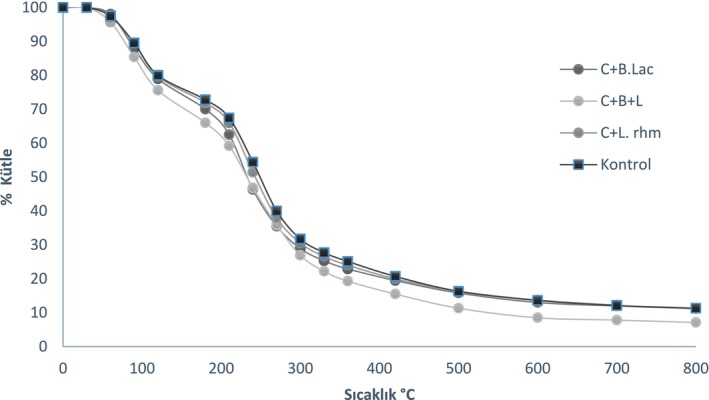
Thermogravimetric analysis (TGA) graph of active film samples.

The thermal decomposition temperatures of the prepared edible film samples and the thermal stability of the polymers were examined using TGA curves. The thermal degradation of the produced biofilms occurred in three main stages. In the initial stage, mass loss up to 100°C can be attributed to the adsorbed moisture on the films, while mass loss starting from 100°C is due to the water present in the edible film structure (Roy and Rhim 2020). In the second stage, the mass loss occurring around 100°C–200°C may be related to the degradation of the glycerol structure. The significant mass loss observed after 200°C also corresponds to the temperature at which alginate begins to decompose (Beghetto et al. 2020; Huntrakul and Harnkarnsujarit 2020). Approximately 35% of the mass was lost in this region. Additionally, after 200°C, mass loss may have occurred due to the decomposition of the organic structure of the probiotic microorganisms. According to similar studies, the organic mass losses of probiotic microorganisms in the film structure are consistent with the TGA curve shown in Figure 4 (Popović et al. 2021; Üçok 2020; Zehir 2017).

### 3.7 Antimicrobial Properties of the Films

The antimicrobial activity of the active film samples against the selected pathogens *Escherichia coli* and *Staphylococcus aureus* is presented in Table 6. As shown in Table 6, the highest antimicrobial effect against *E. coli* was observed with the C‐B+L film samples, which had a zone of inhibition of 45.5 ± 0.71 mm. Similarly, the highest antimicrobial effect against *S. aureus* was also achieved with the C‐B+L film samples. Overall, all probiotic‐containing film samples exhibited antimicrobial activity against both pathogen strains. The C‐B+L film showed the largest inhibition zones against both *Escherichia coli* and *Staphylococcus aureus* . Because these results were obtained from technical replicates of a single production batch, they are presented descriptively and should be interpreted as preliminary observations. The high level of deacetylation of chitosan results in a high‐charged surface due to the free amino groups in the chitosan structure, thereby allowing for a broad antimicrobial interaction with both Gram‐negative and Gram‐positive strains. Many researchers accept the hypothesis that the antimicrobial activity of chitosan arises from its interaction with negatively charged cell membranes via amino groups. This interaction can induce cell membrane lysis or block nutrient flow due to chitosan aggregation on the cell membrane (Tamer et al. 2017; Martins et al. 2014). It is well known that probiotic strains should be able to survive gastrointestinal conditions and adhere to intestinal epithelial cells or mucus. In addition, probiotics may contribute to the maintenance of intestinal barrier function, modulation of immune responses, reduction of serum cholesterol levels, and inhibition of pathogenic microorganisms through the production of antimicrobial compounds and competition for nutrients and adhesion sites (Markowiak and Śliżewska 2017). The antimicrobial effects associated with probiotic bacteriocin production can be influenced by the nature of the edible films/coatings as well as the load of target microorganisms and their sensitivity to the produced bacteriocins (Zoghi et al. 2020; Guimarães et al. 2018). Previous studies have reported that some LABs produce more bacteriocins in polysaccharide‐based environments compared to protein‐based environments and that there is a correlation between bacteriocin production rates and measured antimicrobial effects (de Oliveira et al. 2021). Bacteriocins have shown stronger inhibitory effects against Gram‐positive bacteria, while organic acids act against Gram‐negative bacteria (Bambace et al. 2019). High antimicrobial effects have also been attributed to LAB‐derived peptides, which can interact with target microorganisms through cell membrane components or intracellular targets related to DNA, RNA, or protein synthesis (de Oliveira et al. 2021).

**TABLE 6:** Antimicrobial properties of active film samples.

Pathogenic microorganism	Active film samples			
	K	C‐B.lac	C‐L.rhm	C‐B+L
	Antimicrobial zone diameter (mm)			
*Escherichia coli*	31 ± 1.27	42.5 ± 2.12	32.5 ± 1.76	45.5 ± 0.71
*Staphylococcus aureus*	23 ± 1.41	35.5 ± 1.34	35.5 ± 1.34	44.5 ± 0.71

*Note:* It indicates that the values are expressed as mean ± standard deviation (SD) and provides the definitions of the sample abbreviations (C, C‐B, C‐L, and C‐BL), C: chitosan film without probiotic microorganisms; C‐B: chitosan film containing *Bifidobacterium lactis* ; C‐L: chitosan film containing Lacticaseibacillus rhamnosus GG; C‐BL: chitosan film containing both *Bifidobacterium lactis* and *Lacticaseibacillus rhamnosus* GG.

### 3.8 Quality Characteristics of Film‐Coated Yogurt

The pH values of yogurt samples coated with active surface films, detected on storage days 1, 7, 14, and 21, are shown in Table 7. As seen in Table 7, the pH values of the samples varied between 4.02 and 5.34, with the lowest pH value recorded on the 21st day in yogurt samples coated with C‐B+L film samples. Compared with the control yogurt, the pH values of yogurt samples coated with probiotic‐containing films showed a decreasing trend throughout storage. Similarly, in a study conducted by Ortakci and Sert (2012), the effects of free and encapsulated probiotic *Lactobacillus acidophilus* on yogurt pH during storage were examined. The study concluded that free *Lactobacillus acidophilus* was more effective in reducing yogurt pH compared to encapsulated ones. In another study, Ziar et al. (2012) reported that after 28 days of storage at 4°C, the pH values of yogurts containing free and encapsulated *Bifidobacterium animalis* subsp. *lactis* Bb12 and *Lactobacillus rhamnosus* were similar at the end of the storage period. The results of both studies suggest that bacteria trapped in the film matrix may have restricted the release of the acid they produced. In our study, since probiotic cultures were not used in the control group samples, it is thought that probiotic microorganisms that could be released from the chitosan film matrix led to a decrease in yogurt pH compared to control films. The titratable acidity (TA) values of the samples varied between 0.553% and 0.828% LA. The lowest TA value was detected on the 1st day in control group yogurt samples, while the highest TA value was recorded on the 21st day in yogurt samples coated with C‐B+L film samples. Additionally, the observed increase in %LA values of yogurt samples coated with probiotic‐added active chitosan films was found to be parallel to the decrease in detected pH values. As expected, as the pH values of the obtained products decreased, the titratable acidity (%LA) values increased. Throughout the storage period, the lowest dry matter content of yogurt samples was recorded as 14.94% on the 1st day in control group yogurt samples, whereas the highest dry matter content was found as 19.36% on the 14th day in yogurt samples coated with C‐B+L film samples. Yogurt samples coated with active films generally showed higher dry matter values than the control samples during storage. A similar study conducted by El‐Sayed et al. (2021) explained this situation as being primarily due to the films improving their barrier properties against water vapor permeability and minimizing moisture loss. The highest ash content of yogurt samples during storage was 0.978% on the 14th day in C‐L‐Y group yogurt samples, while the lowest ash content was 0.965% on the 1st day in control group yogurt samples. The ash content of yogurts is known to be related to their NaCl content (Somer 2013). Yogurt samples coated with active films tended to exhibit slightly higher ash contents than the control samples. Since sodium alginate is the sodium salt of alginic acid, it is thought that the amount of mineral matter transferred from the films to the yogurt samples during storage is related to the observed increase in % ash content of yogurt samples. During storage, the density values of yogurt samples ranged between 1.0362 and 1.0394 g/cm^3^. The highest density value was observed on the 7th day in control group yogurt samples, while the lowest density value was also recorded on the 7th day in C‐Y yogurt samples. Density values may vary depending on a specific type of yogurt, its ingredients, and the production process. Yogurt is a product with a density ranging from approximately 1.03 to 1.06 g/cm^3^ (Rinaldoni et al. 2012). The results of our study demonstrated that the density values of yogurt samples coated with active chitosan films fall within this range. As seen in Table 7, the water activity values of the samples varied between 0.9917 and 0.9943, showing fluctuations depending on storage days. The highest water activity values were observed on the 7th and 21st days in C‐B‐Y samples, while the lowest water activity values were recorded on the 21st day in C‐Y samples. Water activity (aw) values are highly significant in food technology since they directly affect the physical, chemical, and microbiological stability of foods, thereby influencing overall food quality (Aktaş and Gölge 2022). The water activity values of the samples were found to be parallel to their dry matter contents, with lower aw values generally detected in yogurt samples with higher dry matter content and higher aw values in samples with lower dry matter content. The serum separation values of the samples ranged between 6.275 and 9.5 mL/25 g. The highest serum separation values were observed in control group samples, while the lowest values were detected in C‐B‐Y group yogurt samples. When the results were evaluated, it was observed that the serum separation values of control group samples increased throughout the storage period due to the effect of starter microorganisms. However, the presence of active film layers on the surface of yogurt samples reduced the serum separation values (Kalantarmahdavi et al. 2022). The presence of active chitosan films on the yogurt surface appears to absorb the water separated from the yogurt gel. Thus, the presence of films can help physically reduce serum separation. Therefore, the higher the water‐holding capacity or porosity of the active film structure, the greater the amount of water absorbed from the yogurt gel (Kalantarmahdavi et al. 2022). It appears that the water absorption capacity of active chitosan films (with or without bacteria) is higher than that of other samples. The water‐holding capacity of chitosan is mainly related to its hydrophilic amino acid content (Kenawy et al. 2007). No apparent differences in serum separation among probiotic‐containing films were observed. The L* values of yogurt samples ranged between 67.85 ± 0.40 and 86.90 ± 0.03, a* values between −1.93 ± 0.38 and 0.11 ± 0.33, b* values between 9.88 ± 0.29 and 19.58 ± 0.94, and ΔE* values between 13.80 ± 0.15 and 29.28 ± 0.32. According to the analysis results, it was found that the color values obtained depended on the type of active film used for yogurt coating. According to the Hunter Lab color scale, L* values measure brightness, −a* represents redness, +a* represents greenness, −b* represents blueness, and +b* represents yellowness. The L* value is obtained by measuring the brightness, whiteness, and dullness index of a food, ranging from 0 to 100, with higher values indicating greater darkness. The a* value determines the position of a food's color on the red‐green scale, while the b* value indicates its position on the blue‐yellow scale (Gençdağ 2017). The ΔE* value expresses color change occurring in foods. When evaluating the color values of yogurt samples obtained in our study, it was observed that the color values of the films used for yogurt coating were related to the color values of the samples. Compared to control group samples, yogurt samples coated with active chitosan films showed a decrease in L* and b* values, while an increase was observed in a* and ΔE* values.

**TABLE 7:** Color values of yogurt samples coated with active chitosan films.

Samples	Storage periods (days)			
	1	7	14	21
Control				
L*	74.94 ± 0.28	78.54 ± 0.74	86.84 ± 0.19	86.90 ± 0.03
a*	−1.93 ± 0.38	−1.81 ± 0.23	−1.85 ± 0.01	−1.70 ± 0.29
b*	10.90 ± 0.11	10.70 ± 0.23	12.91 ± 0.14	13.99 ± 0.30
ΔE*	22.56 ± 0.21	19.20 ± 0.59	13.80 ± 0.15	15.22 ± 0.28
C‐Y				
L*	67.85 ± 0.40	73.60 ± 0.80	79.70 ± 1.09	81.15 ± 0.86
a*	−0.40 ± 0.40	−0.47 ± 1.25	−1.44 ± 0.24	−0.96 ± 0.86
b*	10.97 ± 0.74	11.34 ± 1.25	9.88 ± 0.29	17.27 ± 3.38
ΔE*	29.28 ± 0.32	23.99 ± 7.66	17.78 ± 0.93	20.82 ± 1.74
C‐B‐Y				
L*	72.55 ± 0.34	74.05 ± 2.99	71.52 ± 1.89	84.34 ± 0.71
a*	−1.36 ± 0.38	−0.33 ± 4.91	−1.04 ± 0.27	‐1.80 ± 0.06
b*	9.22 ± 0.34	14.10 ± 4.91	10.42 ± 0.94	11.91 ± 0.06
ΔE*	24.27 ± 0.23	24.83 ± 6.30	25.62 ± 2.03	14.82 ± 0.42
C‐L‐Y				
L*	69.10 ± 0.97	71.37 ± 3.33	79.94 ± 1.73	84.09 ± 0.50
a*	−1.30 ± 0.20	−1.31 ± 1.56	−1.80 ± 0.06	−1.15 ± 0.52
b*	10.30 ± 0.42	15.46 ± 1.56	10.56 ± 0.55	11.47 ± 1.31
ΔE*	27.88 ± 1.05	27.81 ± 3.46	17.88 ± 1.42	14.85 ± 0.71
C‐B+L‐Y				
L*	76.61 ± 1.73	78.14 ± 2.03	75.38 ± 0.74	80.72 ± 0.01
a*	‐1.22 ± 0.22	−0.90 ± 1.51	−1.48 ± 0.01	0.11 ± 0.33
b*	9.91 ± 0.49	16.55 ± 1.51	11.21 ± 0.42	19.58 ± 0.94
ΔE*	20.66 ± 1.43	22.79 ± 0.76	22.27 ± 0.51	22.90 ± 0.66

*Note:* It indicates that the values are expressed as mean ± standard deviation (SD) and provides the definitions of the sample abbreviations (C, C‐B, C‐L, and C‐BL), C‐Y, control yogurt; C‐B‐Y, yogurt coated with *B. animalis* ssp. *lactis* B94‐containing chitosan film; C‐L‐Y, yogurt coated with *L. rhamnosus* GG‐containing chitosan film; C‐BL‐Y, yogurt coated with chitosan film containing both probiotic strains.

**FIGURE 5:**
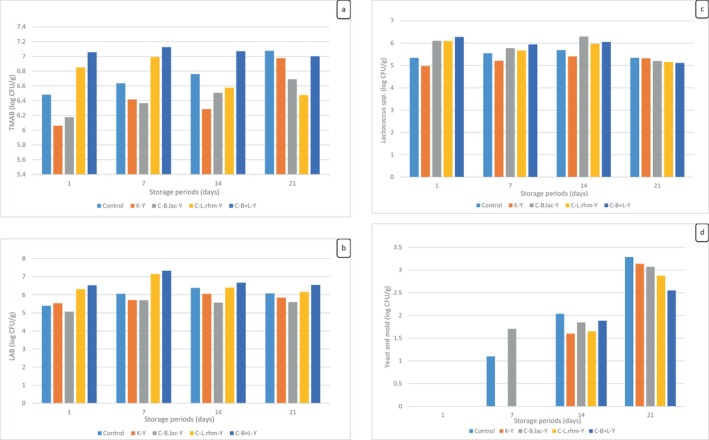
Microbiological properties of coated yogurt samples (log CFU/g); (a) Total mesophilic bacteria counts; (b) Total lactic acid bacteria counts; (c) Total *Lactococcus* spp. counts; (d) Total mold and yeast counts.

The total mesophilic aerobic bacteria (TMAB) counts (log CFU/g) of yogurt samples coated with active chitosan films during a 21‐day refrigerated storage period are shown in Figure 5a. The TMAB count of yogurt samples ranged between 6.06 and 7.125 log CFU/g. The highest TMAB count was detected on the 21st day in C‐B+L‐Y samples, which is believed to be due to the microbial analysis being conducted on yogurt samples along with edible film samples containing encapsulated probiotic microorganisms. The total lactic acid bacteria (LAB) counts (log CFU/g) of yogurt samples during the 21‐day refrigerated storage period are shown in Figure 5b. The LAB count of yogurt samples ranged between 5.07 and 7.33 log CFU/g. It was observed that the LAB count increased throughout the 21‐day cold storage period in all samples. The highest LAB count was detected on the 7th day in C‐B+L‐Y samples. The presence of edible films on the yogurt surface may contribute to the maintenance of starter culture viability by providing a physical protective layer and reducing exposure to external environmental factors (Kasapis 2020). In the present study, active chitosan films appeared to support microbiological stability during storage. However, oxygen permeability was not evaluated; therefore, no direct conclusions can be drawn regarding oxygen limitation or oxygen barrier properties of the developed films. The antimicrobial and nutrient‐rich protective layer on the surface of the yogurt also limits oxygen and prevents spoilage agents, thereby increasing the survival of the starter culture (Delavenne et al. 2013). In this study, it is observed that these layers serve as a protective barrier on the yogurt surface, restricting oxygen and aerobic spoilage microorganisms, and helping to maintain the viability of starter cultures. The total *Lactococcus* spp. counts of yogurt samples ranged between 4.97 and 6.30 log CFU/g (Figure 5c). The highest *Lactococcus* spp. count was recorded on the 14th day in C‐B‐Y samples, while the lowest count was observed on the 1st day in C‐Y samples. Toward the end of the storage period, a decrease in the total *Lactococcus* spp. count was observed in all yogurt samples. These results are consistent with previous studies on microencapsulated probiotic bacteria in yogurt (Mortazavian et al. 2007). Due to the high moisture content of yogurt, this decrease has been attributed to osmotic shock during rehydration. Rehydration of probiotics is a critical step for the revival of cells after dehydration. The rehydration medium can also significantly affect probiotic recovery (Kalantarmahdavi et al. 2020). The total yeast and mold counts of yogurt samples ranged between 0 and 3.28 log CFU/g (Figure 5d). No yeast or mold growth was observed in any sample on the 1st day of storage, whereas the highest yeast and mold count was detected in control group samples on the 21st day of storage. Mostert and Jooste (2002) reported that the total yeast and mold count should be below 10 CFU/g. Additionally, yeast counts at high levels of 10^6^–10^8^ CFU/g have been reported as evidence of spoilage in yogurts (Elsanhoty and Ramadan 2018). In light of this information, it is observed that the total yeast and mold counts of the yogurt samples produced in this study remained within the specified limits.

## 4 Conclusions

In conclusion, microencapsulated probiotic chitosan films were successfully developed and applied as surface coatings for set‐type yogurt. According to the results of this study, chitosan films enriched with microencapsulated probiotic microorganisms can simultaneously enhance the quality and nutritional properties of food products. The viability of probiotic microorganisms after film drying remained high (86.26%–90.94%), indicating that the microencapsulation and film‐forming processes provided effective protection against dehydration stress. The developed films exhibited suitable physicochemical, mechanical, and barrier properties for potential food packaging applications. In this preliminary study, application of the probiotic chitosan films was associated with favorable trends in the physicochemical and microbiological characteristics of yogurt during refrigerated storage.

The novelty of this study lies in the development of microencapsulated probiotic chitosan films and their direct application to set‐type yogurt, together with the comprehensive evaluation of film characteristics and yogurt quality during refrigerated storage. Therefore, based on the findings of this research, active chitosan films enriched with microencapsulated probiotics could be considered promising candidates for application in the food industry as biologically active edible films. The results suggest that these films have potential for use as biologically active edible packaging materials in fermented dairy products and other food applications. However, the viability of *Lacticaseibacillus rhamnosus* GG and *Bifidobacterium animalis* ssp. *lactis* was not determined separately in the final yogurt matrix. Therefore, future studies should employ strain‐specific enumeration methods or molecular techniques to verify the effectiveness of the developed films as probiotic delivery systems. Additional research is needed to evaluate their applicability in other food products.

Future studies should focus on the strain‐specific determination of probiotic microorganisms in the final food matrix using selective microbiological and molecular techniques. In addition, the oxygen and carbon dioxide barrier properties of the developed films should be evaluated through gas permeability analyses to better understand their protective mechanisms. Further research is also needed to investigate the performance of these films in different food systems, optimize film formulations, and evaluate consumer acceptance and industrial‐scale applicability.

A major limitation of the present study is that the yogurt application and antimicrobial activity experiments were conducted using technical replicates derived from a single production batch rather than independent biological or process‐level replicates. Consequently, the findings related to yogurt quality and microbiological characteristics should be considered preliminary. Future studies should include at least three independent production batches of both the edible films and yogurt to evaluate the reproducibility and robustness of the results and to provide stronger evidence for the effectiveness of the developed film system in yogurt preservation under practical production conditions.

## Author Contributions

**Selin Kalkan:** conceptualization, investigation, funding acquisition, writing – original draft, methodology, writing – review and editing, supervision, formal analysis, validation, project administration. **Mustafa Remzi Otağ:** writing – original draft, investigation, validation, writing – review and editing, formal analysis. **Emine Kirkoçoğlu:** investigation, methodology, writing – original draft. **Mehmet Soner Engin:** investigation, writing – original draft, methodology, writing – review and editing, formal analysis.

## Funding

This research has been financially supported by the Scientific Research Projects Coordination Unit of Giresun University (Project No: FEN‐BAP‐C.230123‐05).

## Conflicts of Interest

The authors declare no conflicts of interest.

## Data Availability

Data supporting the findings of this study are available upon request from the corresponding author.
